# *S*-Glutathionylation in Monocyte and Macrophage (Dys)Function

**DOI:** 10.3390/ijms140815212

**Published:** 2013-07-24

**Authors:** Sarah Ullevig, Hong Seok Kim, Reto Asmis

**Affiliations:** 1 Department of Biochemistry, University of Texas Health Science Center San Antonio, 7703 Floyd Curl Drive, San Antonio, TX 78229, USA; E-Mail: ullevigs@uthscsa.edu; 2 Department of Clinical Laboratory Sciences, University of Texas Health Science Center San Antonio, 7703 Floyd Curl Drive, San Antonio, TX 78229, USA; E-Mail: kimhs@uthscsa.edu

**Keywords:** *S*-glutathionylation, monocyte, macrophage, thiol oxidative stress, vascular diseases

## Abstract

Atherosclerosis is a chronic inflammatory disease involving the accumulation of monocytes and macrophages in the vascular wall. Monocytes and macrophages play a central role in the initiation and progression of atherosclerotic lesion development. Oxidative stress, which occurs when reactive oxygen species (ROS) overwhelm cellular antioxidant systems, contributes to the pathophysiology of many chronic inflammatory diseases, including atherosclerosis. Major targets of ROS are reactive thiols on cysteine residues in proteins, which when oxidized can alter cellular processes, including signaling pathways, metabolic pathways, transcription, and translation. Protein-*S*-glutathionylation is the process of mixed disulfide formation between glutathione (GSH) and protein thiols. Until recently, protein-*S*-glutathionylation was associated with increased cellular oxidative stress, but *S*-glutathionylation of key protein targets has now emerged as a physiologically important redox signaling mechanism, which when dysregulated contributes to a variety of disease processes. In this review, we will explore the role of thiol oxidative stress and protein-*S*-glutathionylation in monocyte and macrophage dysfunction as a mechanistic link between oxidative stress associated with metabolic disorders and chronic inflammatory diseases, including atherosclerosis.

## 1. Introduction

Monocyte recruitment and migration are functional characteristics of the innate and adaptive immune system [1,2]. Recruitment and subsequent transendothelial migration, stimulated by chemoattractant cytokines (chemokines), allows monocytes to home to sites of infection, tissue damage, and inflammation. In tissues, monocytes differentiate into macrophages and then release, in a highly regulated manner, numerous cytokines, reactive oxygen species (ROS), and proteases in order to promote inflammatory responses, coordinate the recruitment and activity of other immune cells, and subsequently, to initiate tissue repair and the resolution of inflammation [3]. In addition to their critical roles in immune responses, monocyte-derived macrophages are also associated with the initiation and progression of many chronic inflammatory diseases, including arthritis, multiple sclerosis, and atherosclerosis.

Atherosclerosis, which is responsible for the majority of cardiovascular diseases (CVD) [4], is a chronic inflammatory disease characterized by the progressive accumulation of lipids, cholesterol, calcium, and inflammatory and vascular cells in the vascular wall. Early atherosclerotic lesions are characterized by the accumulation and prolonged persistence of monocyte-derived macrophages in the sub-endothelial space and their transformation into lipid-laden foam cells. Through the release of cytokines and ROS, foam cells and other inflammatory macrophages promote atherosclerotic lesion formation through inflammatory cell recruitment and vascular remodeling, *i.e*., smooth muscle cell migration, extracellular matrix deposition, and platelet activation [3]. Metabolic risk factors for atherosclerosis include hyperglycemia, hyperlipidemia, and hypertension. All of these risk factors are associated with chronic inflammation and oxidative stress [4,5]. We provided evidence that monocyte dysfunction induced by metabolic stress primes monocytes to become hyperresponsive to chemoattractants [6]. Metabolic priming of monocytes is dependent on Nox4-derived hydrogen peroxide (H_2_O_2_) and protein-*S*-glutathionylation [6], the reversible formation of mixed disulfides between glutathione (GSH) and protein thiols [7].

Oxidative stress refers to the imbalance between ROS and cellular antioxidant systems [5]. ROS produced from the mitochondrial electron transport chain as a result of incomplete reduction of superoxide, and nitric oxide synthase (NOS) uncoupling contributes to increased cellular ROS formation. ROS are also produced indirectly as side-products from reactions catalyzed by xanthine oxidase and acyl CoA oxidase in the peroxisome, or directly by the professional ROS producers, NADPH oxidases (Noxs) [8]. ROS is a broad term used to describe various oxygen-containing species, including superoxide radical, hydroxyl radical, nitric oxide, H_2_O_2_, peroxynitrite, and hypochlorous acid [9]. In cells, ROS can be interconverted either enzymatically or non-enzymatically to other species. For example, superoxide is converted to H_2_O_2_ by the enzyme superoxide dismutase, and H_2_O_2_ can decompose to the hydroxyl radical in the presence of iron via the Fenton reaction. Cellular antioxidants consist of enzymes, including superoxide dismutase, catalase, glutathione reductase, glutathione peroxidase, peroxiredoxins, and small molecule redox couples such as glutathione (GSH)/glutathione disulfide (GSSG) and reduced and oxidized nicotinamide adenine dinucleotide phosphate (NADP, NADPH), and ROS scavengers, including vitamins E and C, and uric acid [10], that are designed to counteract increases in ROS levels. ROS produced by NOS uncoupling, the mitochondrial electron transport chain, or by Nox, have all been implicated in the development of cardiovascular diseases, and diabetes and its micro-and macrovascular complications [8,11,12].

The spectrum of oxidative stress varies from physiological to moderate, to severe [13]. Physiological oxidative stress refers to ROS generated under basal cellular conditions or produced in response to a physiological stimulus. Physiological ROS production is generally transient, localized, and results in the oxidative modification of specific cellular targets. For example, ROS production in response to receptor activation by cytokines (tumor necrosis factor-alpha, TNF-α, and interlukin-1, IL-1) or growth factors (platelet-derived growth factor, PDGF, and epidermal growth factor, EGF) involves the oxidation of specific proteins and is required for the activation of downstream signaling cascades (reviewed in [14,15]). Moderate oxidative stress refers to increased or prolonged ROS production that interferes with ROS-mediated signaling pathways and leads to the dysregulation of redox signaling. For example, metabolic stress in THP-1 monocytes induces Nox4, increases ROS production, and hyperactivates cellular pathways, such as the mitogen-activated protein kinase (MAPK) pathway, that are important in monocyte cell adhesion and migration, thereby contributing to chronic inflammation associated with metabolic diseases [6,16]. Chronic over-activation of ROS-mediated pathways can lead to pathological consequences such as atherosclerosis and diabetes [5]. Severe oxidative stress refers to pathological overproduction of ROS production that indiscriminately oxidizes targets, causing permanent damage. Many irreversible oxidative modifications such as the oxidation of thiols to sulfonic acid, and protein carbonylation are the result of high levels of oxidative stress often associated with metabolic diseases including diabetes and cardiovascular disease [17,18].

ROS-induced oxidative modifications can be found on proteins, lipids, deoxyribonucleic acid (DNA), carbohydrates, and various other nutrients and metabolites. Proteins are the largest components of cells and tissues and are thus a major target of ROS [19]. Cysteines comprise 2% of the total amino acid composition of eukaryotic proteins and represent highly conserved and reactive amino acid residues in proteins [20–22]. Cysteines are susceptible to oxidation by ROS generated by a variety of sources and their reactivity increases with decreasing pKa of their thiol [9]. The most abundant cysteine-containing molecule in the cell, however, is GSH, a small three amino acid peptide that contains one cysteine, with a concentration of around 1–11 mM in the cytosol [23]. The ratio of reduced glutathione (GSH) to oxidized glutathione (GSSG) can be used as an estimate of the overall oxidation state of the cellular thiol redox environment [23,24]. The cytosol is considered to be a reducing environment with a cytosolic GSH/GSSH ratio equal to or greater than 100 [23]. Measuring the GSH/GSSG ratio or calculating the overall thiol redox potential based on these GSH/GSSG values, is a method often used to determine the level of (thiol) oxidative stress of cells or tissues. Shifts toward a more oxidizing thiol redox status of cells and tissues (high GSSG, low GSH/GSSG ratio) correlate with disease processes like atherosclerosis and diabetes [25–27].

Thiol oxidative stress refers to the specific oxidation of cysteine and methionine residues in response to environmental stimuli and modulates both the function and oxidation status of proteins [21,28]. Protein thiols have been studied extensively for their role in oxidation/reduction reactions that are critical for a variety of cellular processes, including proliferation, translation and transcription, signal transduction, and apoptosis [21]. H_2_O_2_ produced in response to physiological stimuli, either directly or indirectly via superoxide, has been proposed as a potential mediator of physiological redox signal transduction due to H_2_O_2_’s reactivity with and selectivity for thiols [9,29]. Thiol oxidation products include, but are not limited to, intra- and inter-molecular disulfide bond formation, protein-*S*-glutathionylation, protein-*S*-cysteinylation, sulfenic, sulfinic, or sulfonic acid formation, *S-*nitrosylation, and sulfenylamide [9,30]. Thiol oxidation products have the potential to function as cellular redox signaling proteins, with protein-*S*-glutathionylation emerging as an important redox signaling paradigm [21].

Protein-*S*-glutathionylation can be experimentally measured either as global changes in *S*-glutathionylation or as the *S*-glutathionylation of specific proteins. It is unclear if all reported *S*-glutathionylated proteins actually play a role in redox signaling, especially those formed in conditions of severe oxidative stress. John Mieyal established a set of criteria to determine if a specific *S-*glutathionylation signaling event should be considered a “*bona fide*” redox signaling mechanism (Table 1) [31]. First, *S*-glutathionylation should occur at a discrete site on a protein and change the function of the modified protein. Meeting this first criterion provides evidence for the specificity and the functional importance of the cysteine oxidation. Second, *S*-glutathionylation should occur under physiological conditions, *i.e*., under physiological cellular GSH/GSSG ratios. Third, *S*-glutathionylation should occur within intact cells in response to a physiological stimulus, and elicit a physiological response, such as activation or inhibition of signaling pathways or cellular functions. Instead, many studies have used chemical oxidants like diamide or exogenous H_2_O_2_ at supraphysiological concentrations to promote the formation of *S*-glutathionylated proteins within intact cells or on proteins in cell-free systems. Both criteria #2 and #3 aim to identify physiologically relevant *S*-glutathionylated proteins involved in cellular redox signaling. Fourth, *S*-glutathionylation of specific proteins needs to occur by a defined mechanism for the rapid and efficient formation of *S*-glutathionylated proteins. The exact mechanisms of protein-*S*-glutathionylation in cells are mostly unknown, and many studies still do not address this issue. Potential mechanisms will be discussed below (Section 3). Fifth, *S*-glutathionylation of specific proteins has to occur by a defined mechanism for the rapid and efficient reversal of the *S*-glutathionylated reaction. Glutaredoxin (Grx) is the main cellular enzyme responsible for protein-*S*-deglutathionylation reactions and will be discussed further below (Section 2, Table 1) [31].

Redox signaling events require a high degree of spatial and temporal precision to induce an accurate response. For a thiol, redox signaling mechanism involving ROS to achieve this level of specificity and selectivity, ROS would have to be in close proximity to the target protein. Furthermore, antioxidant enzymes, such as peroxiredoxins, would have to be in close proximity in order to limit the diffusion of ROS and to avoid overt damage and off-target effects [9]. For a redox signaling mechanism to meet Mieyal’s criteria, ROS compartmentalization is necessary to ensure both the specific targeting of thiols and a rapid mechanism for formation of *S*-glutathionylated proteins. Many antioxidant systems, *i.e.*, superoxide dismutase and catalase, have very high reactivity towards superoxide or H_2_O_2_, respectively, and will likely scavenge their substrates before they can react with protein thiols or other protein residues [9,32]. One potential mechanism to achieve both sufficiently high ROS concentration and target specificity would be to selectively recruit NADPH oxidases (Nox), professional ROS producers, directly to redox-sensitive signaling centers within cells [33].

## 2. Nox in Thiol Redox Signaling

The Nox family of enzymes is a group of transmembrane proteins that transport electrons from NAD(P)H across membranes to reduce oxygen to either superoxide or H_2_O_2_ [34]. Nox are important contributors of cellular ROS formation and redox signaling [34–36]. The seven family members, Nox 1-5 and Duox 1-2, differ in their tissue distribution, cellular location, regulatory proteins, and post-translational modifications [34]. All Nox proteins share conserved structural features, including a C-terminal NADPH binding domain, a FAD binding domain, six transmembrane domains, and a heme binding domain. Electrons are transferred from NADPH through flavin adenine dinucleotide (FAD) and heme to oxygen. All Nox proteins are membrane proteins, but their intracellular locations vary between cell type and Nox isoforms, indicating a specialized, targeted function for ROS production. Nox2 is the prime example of this concept as it is located on the plasma membrane with superoxide directionally produced into the extracellular space or phagolysosome, for pathogen killing [34]. Nox1 was found to localize in endosomes and, after hypoxia/reoxygenation injury, produces ROS for the downstream activation of nuclear factor kappa-light-chain-enhancer of activated B cells (NFκB) [33,37]. On the other hand, Nox4 is primarily located in intracellular membranes, such as the endoplasmic reticulum (ER) [38] and the nuclear membrane [39], but also located within the mitochondria [40] and the nucleus [16,41]. Nox4 produces primarily H_2_O_2_ [35,36,42] and, in response to physiological stimuli, appears to specifically oxidize selected cellular proteins [6,34,43]. In addition, Nox proteins have been found to localize to focal adhesion complexes in response to integrin signaling and to produce ROS that inactivate protein tyrosine phosphatases, altering downstream signaling necessary for focal adhesion formation, and cell spreading [33,44]. Recently, recruitment of Nox4 to a membrane scaffold was shown to be necessary for ROS production and activation of the kinase Src, in response to insulin-like growth factor-1 (IGF-1) [45]. Nox proteins can be grouped based on their mode of activation and subsequent ROS production. Nox 1, 2, and 3 require extensive regulatory and adaptor proteins for their activity [34], while Nox4 may require only p22 for activity, although additional activity modulating proteins have been identified. Nox5 and Duox1/2 are activated by calcium [34]. Our lab discovered Nox4 in monocytes and macrophages and demonstrated that Nox4 mediates oxidized low-density lipoprotein (oxLDL)-induced death of human-derived macrophages through the activation of the extracellular-signal-regulated kinase (Erk) and MAPK/ERK kinase 1 (MEK1) pathway [46]. Additionally, Nox4-derived H_2_O_2_ production is increased in metabolically-stressed monocytes and Nox4 mediates increased protein-*S*-glutathionylation, including actin-*S*-glutathionylation, and accelerated chemotaxis induced by metabolic stress [6].

## 3. Protein-*S*-Glutathionylation

Protein-*S*-glutathionylation is a reversible posttranslational modification characterized by the formation of a mixed disulfide bond between protein cysteines and GSH. Originally, increased protein-*S*-glutathionylation in response to cellular ROS production was thought to be a mechanism to protect protein cysteines from irreversible oxidation leading to protein dysfunction or aggregation [23]. More recently, protein-*S-*glutathionylation was proposed to also function as a redox signaling mechanism, as evidenced by its enzymatically reversible nature, its specificity for particular functional cysteines, and its ability to modulate protein functions [7,23]. Most *S*-glutathionylated proteins, however, have not been validated as *bona fide* redox signaling proteins based on Dr. Mieyal’s criteria (Table 1). However, a growing number of *S*-glutathionylated proteins including, but not limited to, actin, MAPK phosphates-1 (MKP-1), p21ras, Fas receptor, sarco/endoplasmic reticulum Ca^2+^-ATPase (SERCA), and protein tyrosine phosphatase 1B (PTP1B), fulfill the majority of these criteria [47].

The mechanisms involved in protein-*S*-glutathionylation are not clear, but several putative mechanisms have been proposed. We do know that proteins can be *S*-glutathionylated through various intermediate oxidation products on protein thiols, including sulfenic acid, *S*-nitrosylation, thiosulfinate, sulfonamide, and thiyl radicals [9,47]. With millimolar cellular concentrations of GSH in the cytosol, most thiol oxidation products readily react with GSH to form *S*-glutathionylated proteins [47]. As a physiological redox signaling mechanism, *S*-glutathionylation of proteins is unlikely to occur due to changes in GSH/GSSG ratios because, in order to induce *S*-glutathionylation by a non-catalyzed disulfide exchange mechanism, the ratio would need to decrease to levels that are not compatible with cell or tissue viability [13,31,48]. There is now evidence for enzymatically catalyzed *S*-glutathionylation involving Grx, glutathione *S*-transferase π, and flavoprotein sulfhydryl oxidase. Enzymatically catalyzed *S*-glutathionylation occurs predominantly in conditions of severe oxidative stress (elevated GSSG levels, low GSH/GSSG ratios) and has not yet been demonstrated as a likely physiological redox signaling mechanisms for protein-*S*-glutathionylation [31]. We have now demonstrated that, in monocytes and macrophages, Nox4-derived H_2_O_2_ is essential for *S*-glutathionylation of proteins involved in monocyte adhesion, migration and recruitment [6,16,49], suggesting that Nox-mediated oxidation of protein thiols and subsequent formation of protein-GSH mixed disulfides may be a physiologically important mechanism for protein *S*-glutathionylation in cells. Grx is the main deglutathionylating enzyme [50], but there is evidence that sulfiredoxin, which primarily reduces sulfenic or sulfinic acids, and certain glutathione *S*-transferase isoforms, can also deglutathionylate proteins [51]. To meet criteria #5 (Table 1), Grx-1 knockdown and overexpression systems *in vitro* and *in vivo* have been used to validate the reversibility of the *S*-glutathionylation reaction of proteins.

Several techniques have been developed to detect *S*-glutathionylated proteins in cells and tissues and distinguish *S*-glutathionylated proteins from other thiol modifications such as inter- and intra-molecular disulfide bond formation, sulfenic acid, *S*-nitrosylation, sulfonamide, and other low molecular weight disulfide partners [21,52]. Detection of *S*-glutathionylated proteins is a challenge because this modification often occurs on low-abundant proteins and often only a fraction of the protein is modified under physiological conditions [9,53]. Many studies therefore utilized non-physiological stimuli, such as diamide, in order to detect measureable levels of *S*-glutathionylated proteins [54–56]. Techniques to identify *S-*glutathionylated proteins fall into one of three categories: (1) detection of global changes in *S*-glutathionylated protein levels in cells or tissues; (2) detection of *S*-glutathionylation on specific proteins and/or cysteines residues; and (3) detection and identification of *S-*glutathionylated proteins using proteomics techniques.

### 3.1. Detection of Global Changes in *S*-Glutathionylated Protein Levels in Cells or Tissues

Changes in total protein-*S-*glutathionylation levels in response to oxidative stress can be measured using a variety of techniques, including colorimetric derivation [57], chromatography [25], Western blot [6,58], and immunohistochemistry [53,59]. Many of these techniques utilize general reductants, such as dithiolthreitol (DTT) or tris(2-carboxyethyl)phosphine (TCEP), to release protein-bound GSH that is then reacted with or coupled to a colorimetric or fluorescent probe [53,58] such as 5,5′-dithiobis-(2-nitrobenzoic acid) (DNTB), *ortho*-phthalaldehyde (OPA) [25] or phenylisothiocyanate [53]. Disadvantages of all these methods include the requirement of large amounts of protein. Colorimetric derivation methods lack specificity as they can also detect other small molecule thiols like cysteine. An antibody directed against GSH bound to proteins (anti-GSH) allows for the quantification of protein-*S*-glutathionylation by Western blot and is the most common way to measure both total *S*-glutathionylated proteins and to detect individual *S*-glutathionylated proteins [53]. The Western blot technique is fairly simple, requires a minimal amount of protein (micrograms), and can be used to detect global *S*-glutathionylation cell lysates and tissues. The specificity of the antibody for individual *S*-glutathionylated proteins are unknown and the antibody’s affinity is likely to vary from protein to protein [53], severely limiting the value of this approach for quantitative analyses. The antibody also primarily detects changes in abundant proteins and therefore low abundant *S*-glutathionylated proteins may not be detected in the total cellular lysate [60]. Although the antibody can be used to detect *S*-glutathionylated proteins in tissues, a more specific technique was developed by Reynaert *et al.* [59] This technique utilizes Grx as a reductant, which specifically reduces *S*-glutathionylated proteins. Once specifically deglutathionylated, proteins are visualized with a thiol-reactive label that is either conjugated with a fluorescent probe or a ligand that is detectable with an antibody [61].

### 3.2. Detection of *S*-Glutathionylation on Specific Proteins and/or Cysteines Residues

To determine whether specific proteins are *S*-glutathionylated, immunoprecipitation techniques are most commonly used. Briefly, a specific antibody against the protein of interest is used to immunoprecipitate the protein, separated by sodium dodecyl sulfate-polyacrylamide gel electrophoresis (SDS-PAGE) and then probed with the anti-GSH antibody to determine if the protein of interest is *S*-glutathionylated [62]. Conversely, *S*-glutathionylated proteins can be immunoprecipitated using the anti-GSH antibody, then probed for the protein of interest [49,62]. However, this low affinity antibody tends to immunoprecipitate only highly abundant proteins. These immunoprecipitation techniques allow for the quantification of individual *S*-glutathionylated proteins, but do not identify the specific cysteine(s) modified with GSH. Alternatively, a number of techniques have been described that utilize a membrane permeable biotinylated-GSH [63,64] (biotinylated GSH ethyl ester, BioGEE), or a biotinylated-GSSG [65] to saturate cells with biotin-labeled GSH. The biotinylated GSH reacts with reactive or oxidized cysteines, *i.e.*, thiolate anion or sulfenic acids, allowing these labeled proteins to be isolated from cellular lysates with the use of streptavidin-coated beads or columns. Enrichment techniques, or the isolation of *S*-glutathionylated proteins from total cellular proteins using biotin and streptavidin, allow for the detection of low abundant modified proteins that might not otherwise be detected amongst high abundant cellular proteins [21]. Limitations of these techniques include failure to identify specific cysteine residues and artificial increases in the cellular GSSG concentrations which can occur during cell labeling, calling into question the physiological relevance of proteins identified via BioGEE and biotinylated-GSSG [58]. To validate if a particular cysteine residue is *S*-glutathionylated, a common technique to assess protein functionality or detect changes in *S*-glutathionylation by Western blot is to mutate the putative reactive cysteines residue(s) to serine residue(s) [49]. Mutation studies can be time consuming depending on how many potential free cysteine residues are present in the protein. Mass spectrometry is used to detect the glutathione adduct(s) on purified or homogenous solutions of *S*-glutathionylated protein, as soft ionization techniques like electrospray ionization (ESI) do not alter the glutathione and protein thiol disulfide bond [53]. Often mass spectrometry is coupled with the aforementioned enrichment techniques to enrich *S*-glutathionylated proteins from a heterogeneous mixture because low-abundance protein modifications are difficult to detect by mass spectrometry (discussed in more detail in Section 3.3).

### 3.3. Detection and Identification of *S*-Glutathionylated Proteins Using Proteomic Techniques

One of the first proteomic studies used for the identification of *S*-glutathionylated proteins utilized radioactive cysteine (l-[^35^S]-cysteine) in order to label intracellular glutathione, then separated the proteins by two-dimensional electrophoresis, and analyzed by mass spectrometry [54]. Although this technique is sensitive, cysteine adducts are labeled and protein synthesis must be inhibited to prevent incorporation of l-[^35^S]-cysteine into cellular proteins [53]. *S*-glutathionylated proteins that contain radioactive GSH can also be useful to identify *S*-glutathionylated proteins by Western blot, utilizing radiography, or by mass spectrometry [53]. Global labeling techniques used to identify individual *S*-glutathionylated proteins couple enrichment techniques with mass spectrometry [54,55,66]. A more recent proteomics technique used to identify reactive cysteines in the redox proteome is referred to as the biotin-switch technique. Briefly, unreacted thiols (free thiols) are alkylated with *N*-ethyl maleimide (NEM) or iodoacetamide (IAM) to prevent further modification [21,55]. Oxidized protein thiols are then reduced using a reductant for a specific thiol oxidative modification. For example, ascorbic acid is used to reduce nitrosylated proteins, while Grx is used to enzymatically reduce *S*-glutathionylated proteins [55]. Limitations of using Grx as a reductant include the cost of the enzyme, the requirement for specific buffer conditions for optimal enzyme activity, the incompatibility of the enzyme with denaturing detergents important in protein solublization, and the “contamination” of the sample with Grx. The newly generated free thiols are then reacted with a labeled thiol-reactive probe. Biotinylated and fluorescently labeled IAM, iodoacetic acid (IAA), or NEM have been used for this technique [55,58,66]. For advantages and disadvantages of NEM or IAM see these references [21,58,67–69]. Labeled proteins are separated through one- or two-dimensional gel separation techniques and are then processed for identification by mass spectrometry. The most common mass spectrometry techniques used to identify *S*-glutathionylated proteins in cell lysates or tissues are matrix-assisted laser desorption/ionization-time of flight (MALDI-TOF) [54,55,65] and liquid chromatography-ESI-tandem mass spectrometry (LC-ESI-MS/MS) [63]. Of these techniques, LC-ESI-MS/MS is the most useful for protein identification because it allows for identification of the peptide sequence.

### 3.4. *S*-Glutationylatined Proteins That Are Established Redox Signaling Molecules

Few *S*-glutathionylated proteins have been established as *bona fide* redox signaling proteins. Ras and PTP1B are two representative examples that have best fulfilled the signaling criteria outlined in Table 1. These are discussed in more detail below.

Ras proteins are a family of small GTPases that modulate downstream signaling pathways important in proliferation, differentiation, and migration, and are known oncogenes [70]. Ras was shown to be *S*-glutathionylated *in vitro* using various reagents, including *S*-nitrosoglutathione (GSNO) [71], GSH and H_2_O_2_, GSSG, and diamide [72–74]. Ras is specifically *S*-glutathionylated on residue C118 which increases ras activity and upregulates downstream signaling pathways, fulfilling the requirement for criteria #1 and #3 [64,75–77]. For example, ras-*S*-glutathionylation on C118 was detected in vascular smooth muscle cells in response to angiotensin II (AngII)-induced hypertropic signaling [75]. In addition, peroxynitrite (ONOO^−^) or oxLDL treatment in bovine aortic endothelial cells (BAECs) resulted in p21ras *S*-glutathionylation, increasing ras activity and subsequent downstream activation of Mek/Erk and phosphatidylinositide-3 kinase (PI3K) and protein kinase B (Akt) signaling [64]. Overexpression of a C118S mutant in BAECs abrogated this response [64]. Also in BAECs, oxLDL treatment increased p21ras *S*-glutathionylation, thereby reducing insulin-induced Akt phosphorylation, which was restored with the C118S mutant [76]. Mechanical strain on cardiac myocytes increased ras-*S*-glutathionylation on C118 and thereby activation of the rapidly accelerated fibrosacroma (Raf)/Mek/Erk pathway [77].

Many of the ras-*S*-glutathionylation studies used Grx-1 overexpression to demonstrate the reversibility of the modification, meeting criteria #5. Using catalase overexpression or an unspecific Nox inhibitor (diphenyleneidonium chloride, DPI), H_2_O_2_ production was found to be necessary for ras-*S*-glutathionylation, although the specific Nox isoform involved was not identified [75]. These studies provide evidence for the functional importance of ras-*S*-glutathionylation for activation of downstream signaling pathways in response to various sources of oxidative stress and mechanical strain.

Protein tyrosine phosphatase 1B (PTP1B) belongs to the larger family of cysteine-based phosphatases (CBP) that contains a nucleophilic and catalytic cysteine that is susceptible to oxidation. [78]. PTP1B is one of the best-studied redox regulated proteins. In response to receptor-mediated growth factor stimulation, Nox-derived H_2_O_2_-induces sulfenic acid formation and *S*-glutathionylation of its catalytic cysteine [78]. PTP1B is the prototypical example of an *S*-glutathionylated protein that meets most of the criteria listed in Table 1 [79–83].

## 4. Protein-*S*-Glutathionylation in Monocyte and Macrophage Dysfunction

The contributions of redox-sensitive mechanisms that regulate monocyte and macrophage function and their importance in the development of atherosclerosis is not well established. Recent evidence suggests that alterations in the intracellular redox environment of monocytes and macrophages not only change their phenotype and functionalities, but also directly affect the development and progression of atherosclerotic lesions. For example, overexpression in mouse macrophages of glutathione reductase (GR), the enzyme responsible for reducing GSSG to GSH, decreases atherosclerosis lesion formation in low-density lipoprotein receptor deficient mice (LDLR^−/−^) mice [84]. Alternatively, overexpressing in mouse macrophages the catalytic domain of glutamate-cysteine ligase (GCL), the rate-limiting enzyme in GSH synthesis, increases total macrophage GSH levels and also reduces atherosclerotic plaque size in apolipoprotein E deficient (APOE^−/−^) mice [85]. Conversely, GCL deficiency in macrophages promotes atherosclerosis; bone marrow transplantation of GCL-deficient bone marrow into APOE^−/−^ mice increased lesion area by 60% [85]. Pharmacological approaches that modulate GSH levels showed very similar results. Peritoneal macrophages isolated from APOE^−/−^ mice fed a diet supplement with L-2-oxo-4-thiazolidin carboylate (OTC), a cysteine precursor, show a significant increase in GSH levels and a reduction in both their lipid peroxide content and their ability to oxidize LDL [86]. OTC-fed mice also showed reduced atherosclerotic lesion formation. In contrast, peritoneal macrophages isolated from APOE^−/−^ mice fed a diet supplemented with buthionine sulfoximine (BSO), an inhibitor of GSH synthesis, showed depleted GSH levels, resulting in increased lipid peroxide content and increased LDL oxidation [86]. Atherosclerotic lesion size was significantly increased in these BSO-fed mice. These studies highlight the importance of maintaining the macrophage thiol redox environment, by providing both a sufficient pool of GSH and maintaining a high GSH/GSSG ratio, in order to prevent atherosclerosis.

The thiol redox status of peritoneal macrophages in diabetic LDLR^−/−^ mice correlates with macrophage chemotactic activity *in vivo*, macrophage recruitment into atherosclerotic lesions and the severity of atherosclerosis in these mice [27]. The same study also showed that, protein-*S*-glutathionylation occurs within aortic lesions in particular macrophages in these atherosclerotic lesions, indicating that hyperglycemia and hyperlipidemia induce thiol oxidative stress in macrophages within atherosclerotic lesions [27]. Collectively, these findings support the hypothesis that metabolic disorders promote thiol oxidative stress in monocytes and macrophages, and that the disruptions of the thiol redox status which lead to monocytes and macrophage dysfunction, appear, at least in part, to be mediated by protein-*S*-glutathionylation.

Monocytes and macrophages play important roles in the initiation and progression of many chronic inflammatory diseases associated with oxidative stress, and protein-*S*-glutathionylation may represent a key mechanistic link between oxidative stress and inflammation. Protein *S*-glutathionylation has been reported in alveolar macrophages (AMs), primary human blood monocytes, human monocyte-derived macrophages, THP-1 human monocytic cell-line, mouse peritoneal macrophages (PMs), and mouse macrophage cell-lines, but only few *S*-glutathionylated proteins have been validated as actual redox signaling proteins. Many of the studies addressing protein-*S*-glutathionylation in monocytes and macrophages measured changes in global *S*-glutathionylation. For example, lipopolysaccharide (LPS)-induced-stress increases protein-*S*-glutathionylation in AMs and promotes an inflammatory response by AMs, which was exacerbated with Grx-1 deficiency [87]. Treatment of human monocyte-derived macrophages with the chemotherapeutic adriamycin dose-dependently increases global protein-*S*-glutathionylation, which correlates with macrophage viability [88]. Additionally, adriamycin-treated mouse PMs show increased protein-*S*-glutathionylation, which correlates with increased ROS formation and decreased macrophage recruitment [88]. Increases in protein-*S*-glutathionylation in oxLDL-treated mouse PMs were associated with increased oxidative stress, as measured by a decrease in the GSH/GSSG ratio, and increased cell death [89]. These studies highlight the importance of protein-*S*-glutathionylation for monocyte and macrophage function, but only few studies have actually identified specific *S*-glutathionylated proteins in monocytes and macrophages (Table 2). Based on Dr. Mieyal’s criteria, many of these proteins do not meet all the characteristics required to be considered a redox signaling protein, many only meeting criteria #1, #2, and #3 (Table 2). Three newly validated proteins, actin, MKP-1, and 14-3-3, meet all the criteria in Table 1. We recently identify Nox4 as the source of ROS responsible for the *S*-glutathionylation of these important signaling molecules in monocytes and macrophages.

Actin and MAPK kinase phosphatase-1 (MKP-1), two key proteins involved in monocyte motility, are *S*-glutathionylated. In response to metabolic and oxidative stress, *S*-glutathionylation of these proteins is increased in THP-1 monocytes, a human monocytic cell-line. Both actin- and MKP-1-*S*-glutathionylation enhance the responsiveness of THP-1 cells to chemoattractants, and accelerate monocyte migration and adhesion, characteristic features of metabolically stressed or “primed” monocytes, a proinflammatory monocyte phenotype associated with metabolic diseases [6,49].

The actin cytoskeleton, through a series of coordinated polymerization and depolymerization events, mediates chemokine-directed cell adhesion and migration necessary for cellular motility [90,91]. Over 100 actin-associated proteins coordinate the dynamic process of cell migration [92]. Recent evidence suggests that, in addition to phosphorylation and dephosphorylation events, actin turnover is redox regulated through protein-*S*-glutathionylation at cysteine 374 [7,93–96]. Interestingly, actin is *S*-glutathionylated even under resting cell conditions in both fibroblasts [94,95] and THP-1 monocytes [6]. In response to epidermal growth factor (EGF) stimulation of fibroblasts, actin is deglutathionylated, increasing actin polymerization and localizing actin filaments (F-actin) to the cell periphery [97].

**Figure 1:**
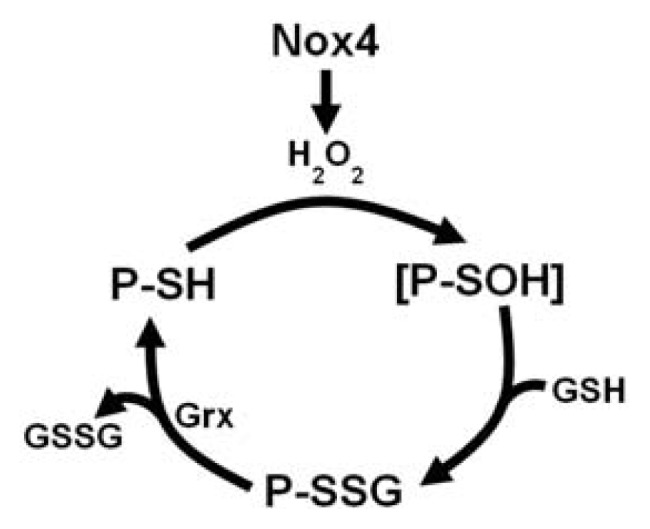
Proposed Mechanism of Nox4 mediated *S*-Glutathionylation in THP-1 Monocytes. Nox4-induced hydrogen peroxide (H_2_O_2_) oxidizes a protein thiol (P-SH) to a putative sulfenic acid intermediate (P-SOH). With the addition of glutathione (GSH), the thiol is further oxidized to form an *S*-glutathionylated protein. Glutaredoxin (Grx) enzymatically reduces the *S*-glutathionylated protein to release oxidized glutathione (GSSG) and the reduced protein thiol (P-SH) [6,31,47].

Our group recently showed that metabolic stress promotes the acceleration of THP-1 monocyte migration in response to chemokines, and that this process we termed “metabolic priming” requires the induction of Nox4. Using both siRNA knockdown and overexpression approaches, Nox4 expression mediates this hyper-reactivity to chemokines at least in part by increasing actin-*S*-glutathionylation. The ratio of filamentous (F) to globular (G) actin is used as an indicator of actin polymerization and actin turnover [98]. Metabolic stress also reduces the F-actin to G-actin ratio in unstimulated THP-1 monocytes and further enhances the monocyte chemoattractant protein-1 (MCP-1)-stimulated decreases in the F-actin to G-actin ratio in these cells, further supporting the notion that actin-*S*-glutathionylation accelerates actin turnover. These data suggest that by increaseing actin dynamics, metabolic stress enables monocyte to more effectively respond to cues from inflamed issues. However, the physiologic significance of this shift to a more proinflammatory monocyte phenotype is not yet clear. Nox4 colocalizes with actin in human monocyte-derived macrophages, providing a mechanism for the specific oxidation of actin thiols by Nox4 [6,16]. In addition, overexpression of Grx-1 significantly reduces metabolic stress-induced actin-*S*-glutathionylation and accelerated migration, indicating the functional relevance and reversibility of actin-*S*-glutathionylation. Our study identified Nox4-derived H_2_O_2_ as the oxidant mediating actin-*S*-glutathionylation, providing a molecular mechanism for this important oxidative modification that regulates actin dynamics and cell motility (Figure 1) [6].

MAPK phosphatases (MKPs), also known as dual-specific phosphatases, have the same catalytic cysteine residue as PTP1B, a well-known site of *S*-glutathionylation and PTP1B inactivation [80]. MKPs counter-regulate and inactivate both ERK and p38, the two principal MAPK pathways mediating MCP-1-induced monocyte adhesion and migration [103,104]. In THP-1 monocytes and primary human blood monocytes, Nox4-derived H_2_O_2_ promotes *S*-glutathionylation of MKP-1, inhibits phosphatase activity, and targets the protein for proteasomal degradation. MKP-1 deficiency in monocytes results in the hyperactivation of MAPK pathways and acceleration of monocyte chemotaxis in response to MCP-1. Furthermore, blood monocytes isolated from diabetic mice showed a 55% reduction of MKP-1 activity compared to non-diabetic mice. Hematopoietic MKP-1-deficiency in atherosclerosis-prone mice mimicked monocyte priming and dysfunction associated with metabolic disorders, increased monocyte chemotaxis *in vivo,* and accelerated atherosclerotic lesion formation [49].

Cofilin, an actin binding protein that promotes actin depolymerization, undergoes phosphorylation and dephosphorylation in response to various extracellular stimuli that also trigger changes in the actin cytoskeleton [105,106]. The slingshot (SSH) family of protein phosphatases has been shown to specifically dephosphorylate and reactivate cofilin by dephosphorylating Ser-3 on cofilin [107,108]. SSH1L is activated by releasing the phosphatase from a regulatory complex with 14-3-3zeta [109]. Recently, we found that metabolic stress promotes the *S*-glutathionylation of 14-3-3zeta, targeting 14-3-3zeta for caspase-dependent degradation [110]. 14-3-3zeta *S*-glutathionylation did not affect binding of 14-3-3zeta to SSH1L, suggesting that degradation and overall loss of total 14-3-3zeta increases the pool of free, unbound SSH1L phosphatase, thereby preventing the phosphorylation and inactivation of cofilin in response to chemokine activation. Increases in free, active SSH1L phosphatase in monocytes resulted in cofilin phosphorylation and activation, and accelerated monocyte chemotaxis in response to MCP-1 [110].

## 5. Conclusions

Redox signaling is evolving as an important signaling paradigm contributing to the regulation of cellular functions. Evidence is emerging that many cysteine-containing proteins are redox regulated, but to date very few have been validated as *bona fide* redox signaling proteins. While some of these proteins meet a several of the criteria set out by Mieyal and coworkers that would allow them to be classified as redox-regulated molecules, most *S*-glutathionylated proteins do not meet all criteria, primarily because no intracellular mechanism has yet been identified that would account for the rapid and effective formation of this oxidative modification. Future studies should focus on verifying all the criteria required before classifying individual *S*-glutathionylated proteins as true redox signaling proteins. Research on protein-*S*-glutathionylation, particularly in monocytes and macrophages, is still in its infancy and future studies on redox sensitive mechanisms are likely to provide important new insight into the (dys)regulation of monocyte and macrophage function, particularly the molecular details that link oxidative stress associated with metabolic disorders to chronic inflammatory diseases. In summary, protein-*S*-glutathionylation is a thiol oxidative modification important in redox signaling and provides an important new mechanistic link between oxidative stress and inflammation.

## Figures and Tables

**Table 1:** Criteria to establish protein-*S*-glutathionylation as a redox signaling mechanism. Adapted from Mieyal, *et al.*, 2008 [31].

*S*-Glutathionylation occurs at a discrete site and changes the function of the modified protein. *S*-Glutathionylation occurs under physiological conditions, *i.e*., relatively high GSH/GSSG ratio. *S*-Glutathionylation occurs within intact cells in response to a physiological stimulus, and elicits a physiological response. There is a rapid and efficient mechanism for the formation of specific *S*-glutathionylated proteins. There is a rapid and efficient mechanism for reversing the *S*-glutathionylation reaction.

**Table 2:** Summary of *S*-Glutathionylated Proteins in Monocytes and Macrophage.

Cell type	Target	Stimulus	Functional importance	Criteria fulfilled	Reference
Rat alveolar macrophage cell-line (NR8383)	Phosphatase and tensin homologue deleted from chromosome 10 (PTEN)	Extracellular adenosine triphosphate (ATP)-induced inflammation	Correlated with activation of PI3K, Akt, ERK1/2, caspase-1 and upregulation of GSH synthesis genes	1, 2, 3	Cruz, *et al.* [99]
Rat alveolar macrophage cell-line (NR8383)	Protein tyrosine phosphatase 1B (PTP1B)	Extracellular adenosine diphosphate (ADP)-stimulation of the respiratory burst	Unknown	1, 2, 3	Rinna, *et al.* [82]
Primary human monocytes	Na-H exchanger isoform 1 (NHE1)	Leptin	Activation of NHE1 which increased intracellular pH	1, 2, 3	Konstantinidis, *et al.* [100]
Primary Mouse peritoneal macrophages (Superoxide Dismutase null)	Caspase-1	ATP, nigericin, or S. aurease supernantant	Decreased caspase-1 activity and decreased IL-1β release	1, 2, 3	Meissner, *et al.* [101]
Mouse macrophage cell-line (J774A.1)	Paraoxonase 1 (PON1)	GSSG	Decreased high-density lipoprotein (HDL)-mediated cholesterol efflux	1, 3	Rozenberg, *et al.* [102]
Human monocytic cell-line (THP-1)	Actin	High glucose (20 mM) and native LDL (100 μg/mL)	Increased actin turnover and accelerated monocyte migration	1,2, 3, 4, 5	Ullevig, *et al*. [6]
Human monocytic cell-line (THP-1)	MKP-1	High glucose (20 mM) and native LDL (100 μg/mL)	Hyperactivation of p38 pathway, increased monocyte migration	1, 2, 3, 4, 5	Kim, *et al.* [49]
